# Supplementary material for: Impact of cryopreservation on immune cell metabolism as measured by SCENITH
Source: Oxf Open Immunol. 2024 Dec 20;6(1):iqae015. doi: 10.1093/oxfimm/iqae015 (PMC11790226; doi:10.1093/oxfimm/iqae015)
Supplement: iqae015_Supplementary_Data [file iqae015_supplementary_data.zip › ffe4f_Supplementary Figures & Tables.pdf]

Supplementary Table 1:

		Antibody			Source	Identifier (Cat. No)
Marker	Staining	Clone	Fluorophore	Concentration		
CD3ε	Extracellular	UCHT1	AF700	1:150	BD Pharmingen	557943
CD45RA	Extracellular	HI100	PE	1:100	BD Pharmingen	555489
CD56	Extracellular	NCAM16.2	PE-CF594	1:100	BD Pharmingen	564849
CD69	Extracellular	FN50	BUV395	1:20	BD Pharmingen	564364
CD4	Extracellular	RPA-T4	BV605	1:50	Biolegend	300555
CD8	Extracellular	SK1	APC-Cy7	1:20	Biolegend	344714
CD14	Extracellular	M5E2	PE-Cy7	1:25	Biolegend	301813
CD16	Extracellular	3G8	BV785	1:250	Biolegend	302045
CD19	Extracellular	HIB19	BUV510	1:100	Biolegend	302241
CCR7	Extracellular	G043H7	BV650	1:20	Biolegend	353233
Granzyme B	Intracellular	QA16A02	AF647	1:50	Biolegend	372219
Perforin	Intracellular	B-D48	PerCP-Cy5.5	1:50	Biolegend	353314
CD25	Extracellular	BC96	BUV737	1:50	eBioscience	367-0259-42
Puromycin	Intracellular	12D10	AF488	1:400	Merck	MABE343-AF488

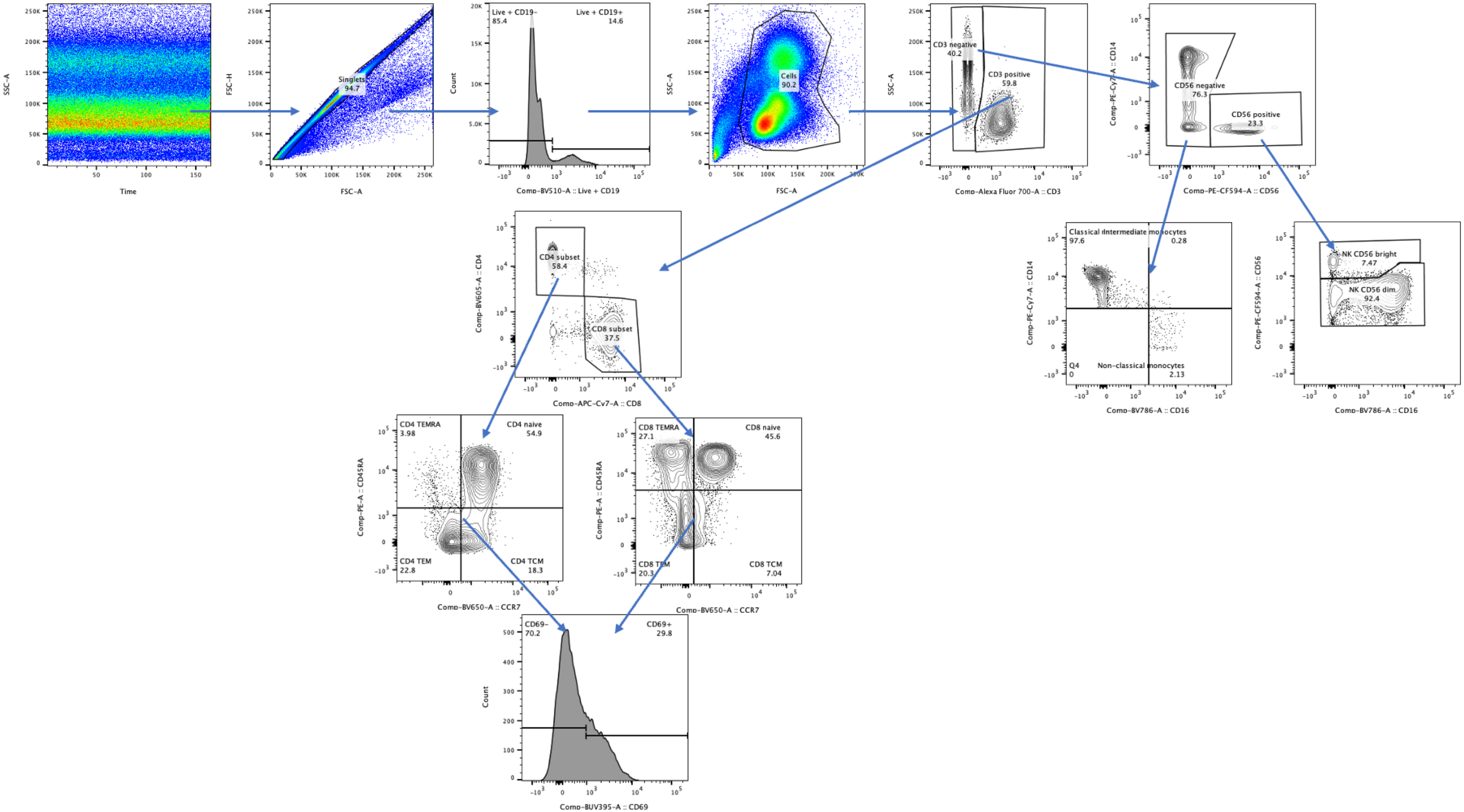
SSC-A
Time
Singlets
94.7
FSC-H
FSC-A
Live + CD19-
85.4
Live + CD19+
14.6
Count
Comp-BV510-A :: Live + CD19
Cells
90.2
SSC-A
FSC-A
CD3 negative
40.2
CD3 positive
59.8
SSC-A
Comp-Alexa Fluor 700-A :: CD3
CD56 negative
76.3
CD56 positive
23.3
Comp-PE-Cy7-A :: CD14
Comp-PE-CF594-A :: CD56
CD4 subset
58.4
CD8 subset
37.5
Comp-BV605-A :: CD4
Comp-APC-Cy7-A :: CD8
Classical
97.6
Intermediate monocytes
0.28
Q4
0
Non-classical monocytes
2.13
Comp-PE-Cy7-A :: CD14
Comp-BV786-A :: CD16
NK CD56 bright
7.47
NK CD56 dim
92.4
Comp-PE-CF594-A :: CD56
Comp-BV786-A :: CD16
CD4 TEMRA
3.98
CD4 naive
54.9
CD4 TEM
22.8
CD4 TCM
18.3
Comp-PE-A :: CD45RA
Comp-BV650-A :: CCR7
CD8 TEMRA
27.1
CD8 naive
45.6
CD8 TEM
20.3
CD8 TCM
7.04
Comp-PE-A :: CD45RA
Comp-BV650-A :: CCR7
CD69-
70.2
CD69+
29.8
Count
Comp-BUV395-A :: CD69

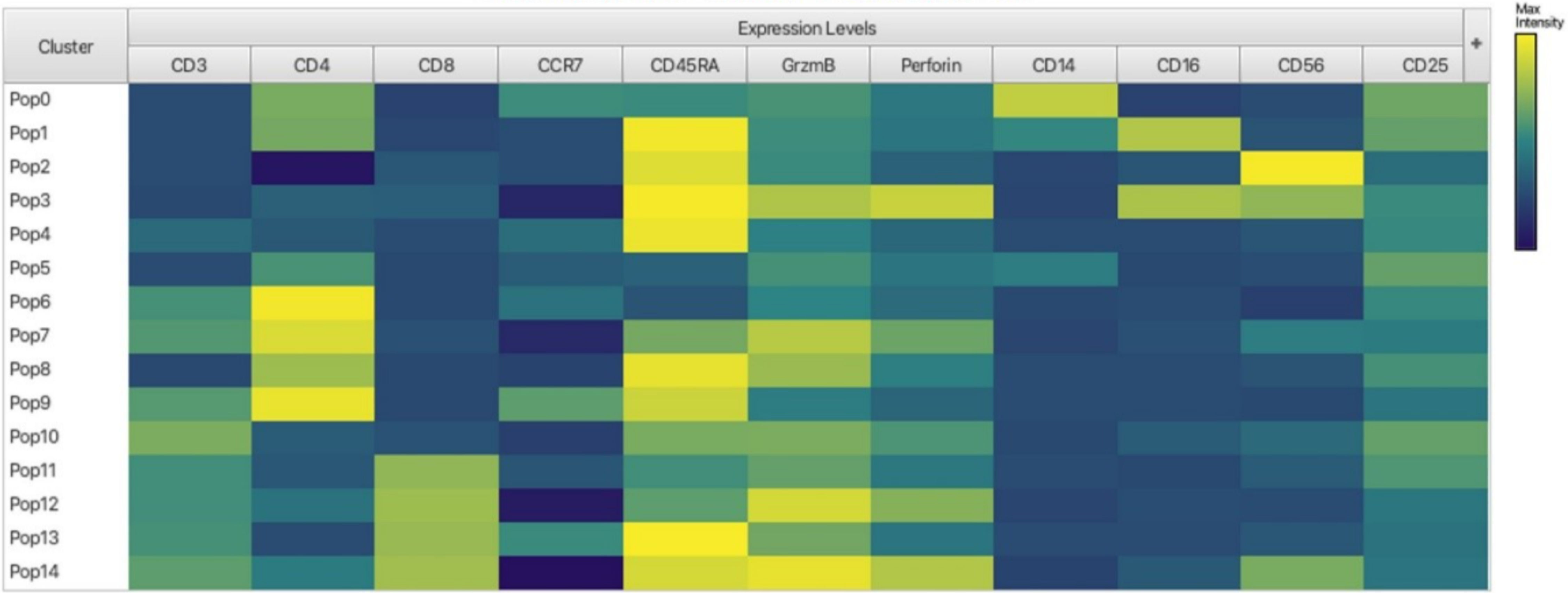

Cluster
Expression Levels
CD3
CD4
CD8
CCR7
CD45RA
GrzmB
Perforin
CD14
CD16
CD56
CD25
Pop0
Pop1
Pop2
Pop3
Pop4
Pop5
Pop6
Pop7
Pop8
Pop9
Pop10
Pop11
Pop12
Pop13
Pop14
Max Intensity
